# Comparison between New-Onset and Old-Diagnosed Type 2 Diabetes with Ketosis in Rural Regions of China

**DOI:** 10.1155/2016/3010243

**Published:** 2016-02-04

**Authors:** Shichun Du, Xia Yang, Degang Shi, Qing Su

**Affiliations:** ^1^Department of Endocrinology, Xinhua Hospital, Shanghai Jiaotong University School of Medicine, Shanghai 200092, China; ^2^Department of Endocrinology, Baoshan People's Hospital, Yunnan 678000, China

## Abstract

*Objectives*. Type 2 diabetes (T2D) with ketosis was common because of late diagnosis and lacking adequate treatment in rural regions of China. This study aimed to provide the data of T2D with ketosis among inpatients in a south-west border city of China.* Methods*. Data of 371 patients of T2D with ketosis who were hospitalized between January 2011 and July 2015 in Baoshan People's Hospital, Yunnan, China, were analyzed. New-onset and old-diagnosed T2D patients presenting with ketosis were compared according to clinical characteristics, laboratory results, and chronic diabetic complications.* Results*. Overall, the blood glucose control was poor in our study subjects. Male predominated in both groups (male prevalence was 68% in new-onset and 64% in old-diagnosed groups). Overweight and obesity accounted for 50% in new-onset and 46% in old-diagnosed cases. Inducements of ketosis were 13.8% in new-onset and 38.7% in old-diagnosed patients. Infections were the first inducements in both groups. The prevalence of chronic complications of diabetes was common in both groups.* Conclusions*. More medical supports were needed for the early detection and adequate treatment of diabetes in rural areas of China.

## 1. Introduction

The prevalence of type 2 diabetes (T2D) in rural regions is rising rapidly and has been a great challenge to the local health care programs [1]. In China, large parts of diabetic individuals lived in rural areas, where resources to screen and treat diabetes and its complications were limited [1–3]. Patients with diabetes usually delayed their adequate treatment due to late screen and diagnosis. Ketosis was frequently presented in these patients. So far, the situations of type 2 diabetes presenting with ketosis prior to admission in the south-west rural region were unclear.

Ketosis is a symbol of acute disorder of glucose and lipids metabolism in diabetic patients. Ketosis or ketoacidosis has been regarded as trait of type 1 diabetes (T1D). However, patients with T2D were susceptible to ketosis or ketoacidosis under extreme hyperglycemia. Because of backward in economy, quite a few patients did not get duly treatment until severe hyperglycemia or diabetic complications occurred. T2D patients with ketosis were frequently seen at clinic in rural areas. Our present study was to investigate the prevalence and characteristics of T2D presenting with ketosis in rural areas aiming to suggest more efforts for the prevention and control of diabetes in rural areas of China.

## 2. Methods

We performed a retrospective analysis of T2D with ketosis in patients admitted to the Endocrinology Department of Baoshan People's Hospital of Yunnan Province between January 2011 and July 2015. Overall, data of 391 T2D presenting with ketosis inpatients aged 12 years or older were enrolled. Nine patients were excluded because of surgery, pregnancy, trauma, secondary diabetes, or pancreatic exocrine diseases. 11 patients were excluded because of complicated with unconsciousness. Thus, the other 371 patients (245 males and 126 females) were allocated to the analysis. According to the diabetic duration, patients were divided into new-onset or old-diagnosed group. New-onset group included those with new diagnosed diabetes or no more than 6 months after onset. Old-diagnosed group were patients of previously diagnosed T2D presenting with ketosis at this admission. Diabetes were defined in accordance with diagnostic criteria of American Diabetes Association [4]. T2D was diagnosed if patients had clinical and metabolic features including overweight or obesity, more than 40 years age at onset, and obvious diabetes family history in combination with preserved *β*-cell function (fasting C-peptide of more than 0.33 nmol/L) and negative *β*-cell autoantibodies. T2D with ketosis was diagnosed if patients met the criteria of T2D and had positive urinary ketone body results this time on admission. Urinary ketone body positive was diagnosed if urinary acetoacetate increased over 15 mg/dL by using sodium nitroprusside method. Acidosis was diagnosed if an arterial pH of less than 7.35 and/or blood bicarbonate level of less than 15 mmol/L in the context of hyperglycemia and ketonuria was found. Overweight and obesity were defined if body mass index (BMI) ≥ 24, which was set forth by Chinese guidance of overweight and obesity in adults [5]. Data of demographic and clinical characteristics including age, gender, height, weight, BMI, blood pressure, and diabetic family history were collected. Laboratory parameters including plasma glucose, HbA1c, C-peptide, total cholesterol, and triglycerides were recorded. Blood samples were performed at the time on admission after an overnight fast. The chronic diabetic complications including peripheral neuropathy, retinopathy, diabetic foot, and persistent microalbuminuria that were evaluated by trained physicians during the hospitalization.

All of the data were analyzed by JMP 9.0 (SAS Institute, Cary, NC). The ANOVA or Mann-Whitney test analysis was used to evaluate the differences in continuous variables. Chi-squared statistical analysis was used for categorical variables. Continuous data in the text and tables were expressed as means ± SD. Categorical variables were expressed by either absolute numbers or percentages. Statistical significance was defined as two-tailed *P* values <0.05.

## 3. Results

Table 1 showed the clinical characteristics of the new-onset and old-diagnosed T2D with ketosis patients. Among the total 371 subjects, 211 of the patients (57%) were classified as new-onset, while 160 (43%) were old-diagnosed T2D with ketosis. The subjects of new-onset group had an average duration of 1.7 months with weight loss of 7.3 kg, while the old-diagnosed group had an average diabetic duration of about 6.3 years with weight loss of 9.2 kg. Subjects of new-onset group were younger (47 ± 12 versus 53 ± 13 yr, *P* = 0.001) and more overweight (50% versus 46%, *P* = 0.009) than those old-diagnosed T2D with ketosis. Family history of diabetes was similar (*P* = 0.79) between new-onset (35%) and old-diagnosed (34%) group. The blood pressure of the analyzed subjects was not significantly different between two groups.

Table 2 showed the biochemical characteristics of the new-onset and previously diagnosed T2D patients presenting with ketosis. Fasting plasma glucose was significantly elevated in both new-onset (17.0 ± 6 mmol/L) and old-diagnosed (16.4 ± 7 mmol/L, *P* = 0.49) group. 2 h plasma glucose was also similarly elevated (*P* = 0.86) between new-onset (24.2 ± 8 mmol/L) and old-diagnosed (24.1 ± 9 mmol/L) group. Fasting C-peptide levels were similar (*P* = 0.60) between new-onset (0.69 ± 0.11 nmol/L) and old-diagnosed (0.58 ± 0.17 nmol/L) group. However, 2 h C-peptide was significantly higher (*P* = 0.02) in new-onset (2.58 ± 1.1 nmol/L) than old-diagnosed (1.26 ± 0.9 nmol/L) group. The hemoglobin A1c (HbA1c) and fructosamine were not different between the two groups. More patients in the old-diagnosed group (19.4%) suffered from acidosis than new-onset group (6.6%). Total cholesterol (5.9 ± 1.7 versus 4.9 ± 1.8 mmol/L, resp., in new-onset and old-diagnosed group, *P* < 0.0001) and triglyceride (4.4 ± 4.0 versus 3.7 ± 4.2 mmol/L, resp., in new-onset and old-diagnosed group, *P* = 0.004) were significantly higher in new-onset than in old-diagnosed group.

**Figure 1:**
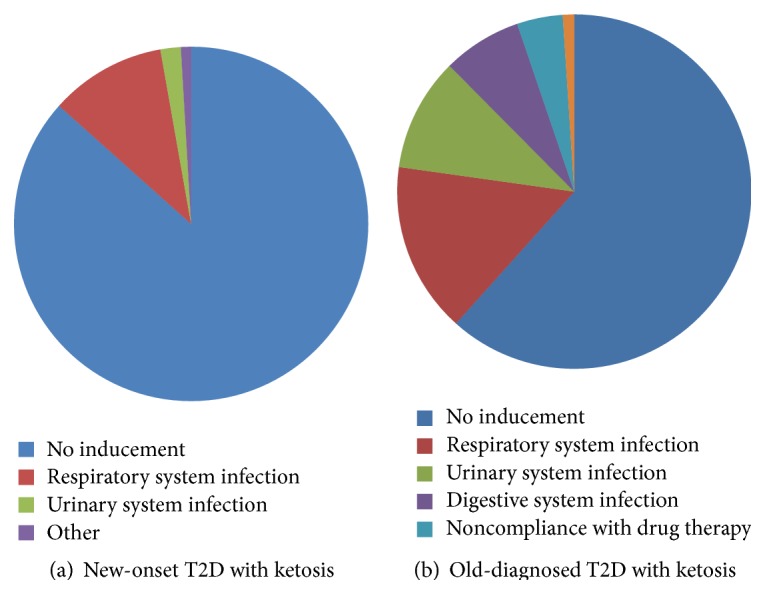
Potential factors precipitating ketosis in T2D patients.

Figure 1 showed the potential factors precipitating ketosis in T2D patients. Those new-onset patients showed a lower incidence of predisposing factors than previously diagnosed T2D patients presenting with ketosis (13.8% versus 38.7%, *P* < 0.001). The potential triggers for subjects in T2D with ketosis were varied. Among them, infections were predominant in both groups. The prevalence of respiratory system infection was 10.3% in new-onset and 14.9% in old-diagnosed group. Urinary system infection was 1.8% in new-onset and 9.9% in old-diagnosed group.

**Figure 2:**
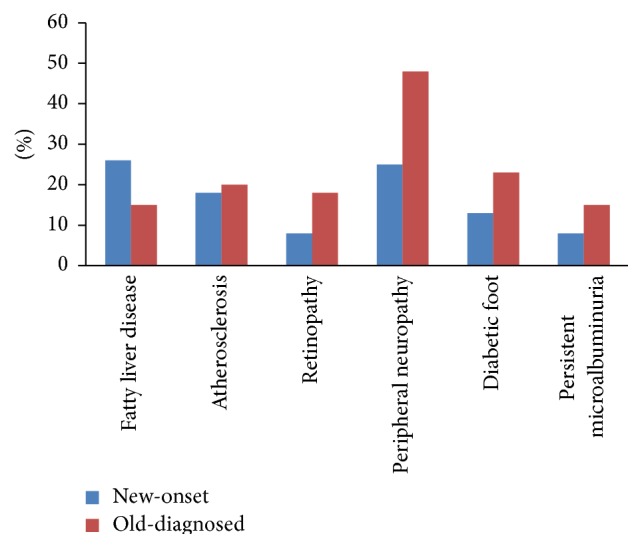
Prevalence of diabetic chronic complications in new-onset and old-diagnosed T2D with ketosis.

Figure 2 showed the prevalence of chronic diabetic complications in new-onset and previously diagnosed T2D with ketosis. The patients with new-onset had higher (*P* < 0.0001) prevalence of fatty liver disease (26.2%) compared with those of old-diagnosed (15.1%) group, while the prevalence of most chronic complications was higher in old-diagnosed group than new-onset group, including retinopathy (8.1% versus 17.8%, resp., in new-onset and old-diagnosed group, *P* < 0.0001), peripheral neuropathy (25.3% versus 47.6%, resp., in new-onset and old-diagnosed group, *P* < 0.0001), diabetic foot (13.3% versus 23.2%, resp., in new-onset and old-diagnosed group, *P* < 0.0001), and persistent microalbuminuria (8.2% versus 15.1%, resp., in new-onset and old-diagnosed group, *P* < 0.0001). No significant differences (*P* = 0.45) in atherosclerosis were found in new-onset (18.0%) and old-diagnosed (20.1%) group.

## 4. Discussion

In this study, we showed the characteristic of T2D with ketosis in rural areas of south-west part of China. It was concluded from the study that the control of T2D was poor in rural area, as reflected not only by extremely elevated HBA1c and plasma glucose performed once on admission but also by high prevalence of chronic diabetic complications evaluated during hospitalization. There were limited data about the prevalence of ketosis in type 2 diabetes population. In a study of 134 Chinese patients with severe hyperglycemia, there were 24 patients diagnosed with ketosis (17.9%) and 6 patients diagnosed with ketoacidosis (4.5%) [6]. The data indicated the high prevalence of ketosis in T2D with severe hyperglycemia.

Subjects of new-onset and old-diagnosed T2D with ketosis group had similarities and differences at clinical manifestations. Consistent with previous studies, we found a predominance of male proportion in the study subjects [7–11]. This prevalence was more obvious in those of new-onset group. The underlying mechanisms of male predominance in T2D with ketosis were unknown and needed further investigation. Differences of body fat distribution and sex hormone were supposed to be factors contributing to the gender difference [12, 13].

In our study, the prevalence of hyperlipidemia was high in both new-onset and old-diagnosed subject, which were true with many previous studies [11, 14–16]. Some investigators have reported that acute elevation of free fatty acid levels in the circulation increased insulin resistance and impaired *β*-cell function [17, 18]. In our study subjects, the prevalence of hyperlipidemia was 60% in subjects of new-onset group and 29% in old-diagnosed group. We analyzed our data and found that hyperlipidemia was common in the overweight diabetic subjects. BMI of the enrolled patients positively correlated with both triglyceride and total cholesterol level (data not shown). The phenomenon further confirmed the view that hyperlipidemia played substantially roles underlying trigger of ketosis especially in new-onset diabetes. Therefore, interventions for prevention and treatment of overweight and hyperlipidemia were essential in T2D patients.

Infections were the most common precipitating factors for ketosis in T2D patients, which was an important point distinguishing T2D from T1D. In patients with T1D, noncompliance with insulin therapy was considered as first reason for precipitating ketosis [14, 19]. In our old-diagnosed T2D subjects, varied infections accounted for about 40% of the total precipitating factors, indicating that infections were the important precipitating factors leading to ketosis. However, in new-onset ketosis subjects, most cases were nonprovoked ketosis. The discrepancy in prevalence of precipitating factors might indicate the complexity and heterogeneity of T2D with ketosis. More clinical epidemiological surveys need to be conducted to clarify the heterogeneity in T2D with ketosis.

In our study subjects, patients had a higher incidence of diabetic chronic complications, especially in those previously diagnosed T2D patients. It was shown by literatures [20] that those who came for regular follow-up diabetic treatment had a lower incidence of retinopathy and nephropathy compared to those who had irregular follow-up. Since T2D and most of the chronic complications were silent at first, patients usually did not come to the clinic for routine examination until they developed severe complications of diabetes in rural areas [20]. As a result, most patients were complicated with severe chronic complications at their fist diabetic diagnosis. However, late treatments of diabetes and its complications were often associated with higher costs to the affected patient [1, 20]. Therefore, it was reasonable to suggest detection and adequate treatment at an earlier stage, by which the excess cost due to treatment of diabetes complications might be minimized.

There were some limitations to the present study. First, we did not perform plasma ketosis test which was more sensitive and specific in detecting ketosis; however, urinary ketone body measurements were widely acceptable in hospitals for diagnosing ketosis. Second, we did not measure the body fat distribution which was a potential and important parameter for explaining gender difference in T2D with ketosis. Third, the single-center study restricted our ability to assess the overall characteristics of T2D with ketosis in rural areas. Further large cohorts and prospective follow-up studies were needed to clarify the characteristics of T2D with ketosis in south-west rural parts of China.

In summary, the study shows the characteristics of T2D with ketosis in adults of rural areas in south-west part of China. T2D with ketosis is a severe disorder of glucose and lipids metabolism and prone to acquire chronic complications. Thus, there is an urgent and important need for the implementation of effective screening, health knowledge promotion, and control of diabetes.

## Figures and Tables

**Table 1:** Clinical characteristics of the new-onset and old-diagnosed T2D with ketosis patients.

	T2D with ketosis		
	New-onset	Old-diagnosed	*P*
Subjects (total %)	211 (57%)	160 (43%)	—
Duration (months)	1.73 ± 2.5	75.36 ± 63.2	—
Weight reduction (kg)	7.31 ± 4.45	9.23 ± 6.34	0.03
Age (yr)	47 ± 12	53 ± 13	0.001
Gender (male %)	68	64	0.15
Diabetic family history (%)	35	34	0.79
Height (cm)	163 ± 9	160 ± 10	0.01
Weight (kg)	66 ± 14	62 ± 15	0.04
Body mass index (kg/m^2^)	25 ± 4	24 ± 5	0.28
Overweight or obese (%)	50	46	0.009
Systolic pressure (mmHg)	119 ± 20	122 ± 21	0.25
Diastolic pressure (mmHg)	79 ± 12	78 ± 13	0.61

**Table 2:** Laboratory results of the new-onset and old-diagnosed T2D with ketosis patients.

	T2D with ketosis		
	New-onset	Old-diagnosed	*P*
Fasting glucose (mmol/L)	17.0 ± 6	16.4 ± 7	0.49
Postprandial 2-hour glucose (mmol/L)	24.2 ± 8	24.1 ± 9	0.86
Fasting C-peptide (nmol/L)	0.69 ± 0.11	0.58 ± 0.17	0.60
Postprandial 2-hour C-peptide (nmol/L)	2.58 ± 1.1	1.26 ± 0.9	0.02
Hemoglobin A1c (%)	13.2 ± 3.2	13.7 ± 4.1	0.34
Fructosamine (mmol/L)	4.05 ± 0.9	3.61 ± 0.8	0.11
Ketoacidosis (%)	6.6	19.4	<0.0001
Total cholesterol (mmo/L)	5.9 ± 1.7	4.9 ± 1.8	<0.0001
Triglyceride (mmo/L)	4.4 ± 4.0	3.7 ± 4.2	0.004
Serum creatinine (*μ*mol/L)	84.6 ± 31	87.1 ± 37	0.28
Serum albumin (g/L)	41.4 ± 7	40.1 ± 6	0.35
